# Bilaterale subretinale Flüssigkeit und pigmentierte Ablagerungen bei einer jungen Patientin

**DOI:** 10.1007/s00347-021-01389-2

**Published:** 2021-04-23

**Authors:** D. Loos, C. P. Lohmann, Ines Lanzl, M. Maier

**Affiliations:** Klinik und Poliklinik für Augenheilkunde am Klinikum rechts der Isar, Ismaninger Str. 22, 81675 München, Deutschland

## Anamnese

Eine 35-jährige Patientin, die wegen eines multiplen Myeloms (MM) vom Typ Bence-Jones-Leichtketten-Kappa (LK-kappa) in stationärer internistischer Behandlung war, wurde uns zur Beurteilung einer plötzlich aufgetretenen, beidseitigen Visusminderung vorgestellt.

Neben dem MM litt die Patientin an einer Anämie, Niereninsuffizienz, sekundärem Hyperparathyreoidismus, arterieller Hypertonie, latenter Hyperthyreose und Hyperlipoproteinämie.

Die aktuelle Medikamentenanamnese umfasste Bisoprolol, Amlodipin, Colecalciferol, Eisen, Chlortalidon, Moxonidin, Clonidin, Natriumhydrogencarbonat, Atorvastatin, Valaciclovir, Pantoprazol, Polysulfonsäure, Enoxaparin, Trimethoprim/Sulfamethoxazol, Allopurinol und Levothyroxin. Vier Zyklen einer Bortezomib-Therapie hatten in Kombination mit Steroidtherapien zu einer kompletten Remission des MM geführt. Nach Apherese, Leukaphärese und Stammzellmobilisierung war 2 Wochen vor dem Beginn der ophthalmologischen Beschwerden Melphalan verabreicht und eine periphere Stammzelltransplantation vorgenommen worden.

## Klinik

**Figure 1:**
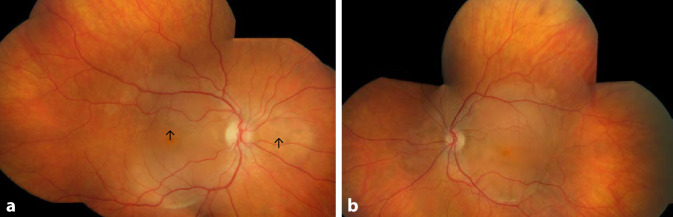

Die bestkorrigierte Sehschärfe der Patientin lag bei der Erstvorstellung bei 0,4 rechts und 0,3 links, der intraokulare Druck war im Normbereich. Der vordere Augenabschnitt zeigte sich regelrecht, die Linse erschien klar. Am Augenhintergrund waren seitenähnliche Befunde mit einer Abhebung der neurosensorischen Netzhaut, intraretinale zystoide Veränderungen und pigmentierte Ablagerungen peripapillär und in der Makula zu erkennen. Die Gefäße erschienen klinisch regelrecht, die periphere Netzhaut anliegend (Abb. 1a, b).

## Weitere Diagnostik

**Figure 2:**
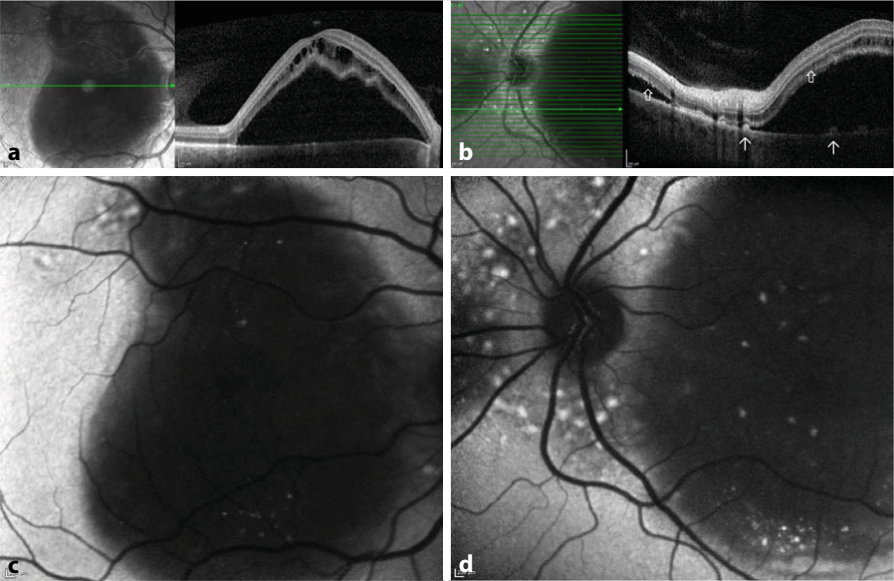

Es erfolgte die Fotodokumentation (Abb. 1). Die optische Kohärenztomographie (OCT) bestätigte die subretinale Flüssigkeit mit zystoiden intraretinalen Strukturauflockerungen im Bereich der äußeren Körnerschicht. Zudem waren kleine, hyperreflektive intra- und subretinale Exsudate und ebenso große hyperreflektive Veränderungen oberhalb des retinalen Pigmentepithels im Bereich der klinisch sichtbaren pigmentierten Ablagerungen erkennbar. Zudem erschienen die Außensegmente elongiert und heterogen (Abb. 2a, b). Die Fundusautofluoreszenz (FAF) stellte sich analog zur Nahinfrarotaufnahme dar (Abb. 2c, d).

**Figure 3:**
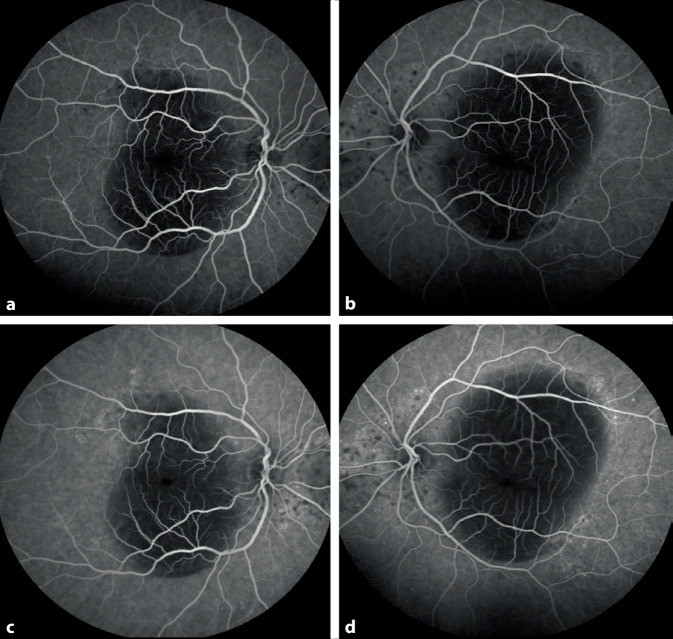

Die Frühphase der Fluoreszeinangiographie zeigte eine Hypofluoreszenz der Makula durch Abschattung der subretinalen Flüssigkeit und ebenso Hypofluoreszenzen durch die Abschattung der großen Ablagerungen. In der Spätphase wurden in der mittleren Peripherie mikroangiopathische Veränderungen mit Mikroaneurysmen und perivasalen Leckagen deutlich (Abb. 3).

## Wie lautet Ihre Diagnose?

Ein Diabetes mellitus war nicht bekannt, die Blutzuckerlage der Patientin war zu keinem Zeitpunkt entgleist. Ein Hypertonus lag zwischenzeitlich vor, der Blutdruck war aber zum Zeitpunkt des Beschwerdebeginns gut eingestellt und kontrolliert. Die Medikamentenliste der Patientin war umfangreich, jedoch sind die beschriebenen Veränderungen bisher nicht als Nebenwirkung der verabreichten Therapeutika bekannt.

Die Literaturrecherche ergab, dass eine seröse Makulaabhebung mit intra- und subretinalen bzw. epipigmentepithelialen Ablagerungen als seltene Manifestation des multiplen Myeloms dokumentiert ist. Die Erkrankung kann als „Gammopathie-Makulopathie“ oder „Paraproteinämie-Makulopathie“ bezeichnet werden [1, 2]. In einem Fallbericht aus diesem Jahr im *BMC Ophthalmology* wurde das Krankheitsbild unserer Patientin erstmals veröffentlicht und deskriptiv als „progressive chorioretinale Beteiligung bei einem Patienten mit Leichtketten-Amyloidose“ beschrieben [3], wobei im hier beschriebenen Fall – anders als im englischen Bericht – zum Zeitpunkt der okulären Beteiligung bereits eine Remission eingetreten war (LK-kappa 29,18 mg/l, initial 895,5 mg/l).

## Therapie und Verlauf

**Figure 4:**
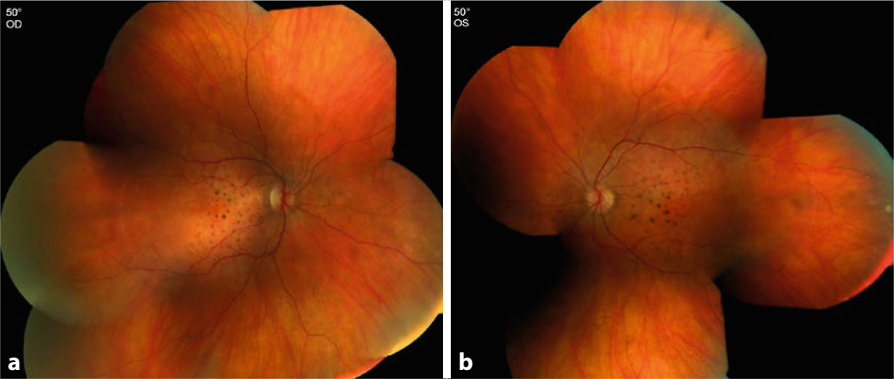

Bei einer kurzfristigen Kontrolle der Patientin ohne Therapie wurde die Sehschärfe schlechter (RA 0,2, LA 0,1), sodass wir uns zu einer gewichtsadaptierten Steroidtherapie mit 1 mg/kgKG Prednisolon p.o. für 5 Tage und folgend Reduktion des Steroids über 4 Wochen entschieden. Hierunter besserten sich die subjektive und objektive Sehschärfe auf 0,7 rechts und 0,8 links. Im Bereich der Makula demaskierten sich zunehmend pigmentierte Ablagerungen (Abb. 4a, b).

**Figure 5:**
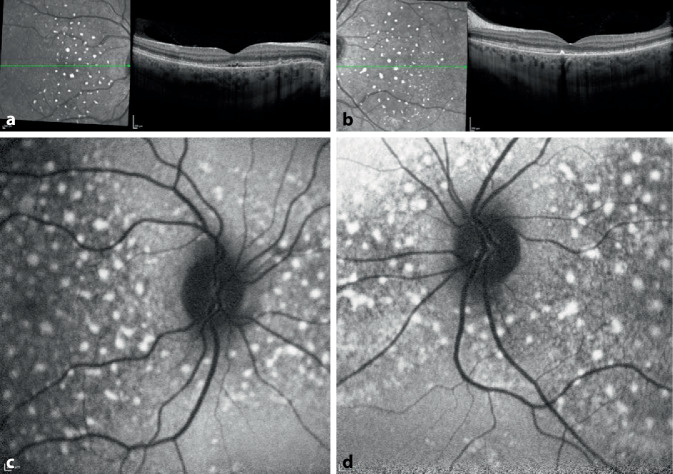

Im OCT war die Abnahme der subretinalen Flüssigkeit nachvollziehbar, in der ellipsoiden Zone, im äußeren Segment der Photorezeptoren und in der Interdigitationszone wurden Unregelmäßigkeiten sichtbar. Die zahlreichen Ablagerungen blieben unverändert. Neu messbar war ein Pachychoroid mit einer choroidalen Dicke von 500 µm (Abb. 5a, b). Die FAF zeigte neben den hyperfluoreszenten Ablagerungen eine körnige Hintergrundfluoreszenz des retinalen Pigmentepithels.

## Diskussion

Das multiple Myelom ist eine maligne lymphoproliferative B‑Zell-Erkrankung, die funktionslose Immunglobuline oder Immunglobulinleichtketten produziert [4]. Neben dem Knochenmark treten auch extraskeletale Manifestationen, in seltenen Fällen auch am Auge, auf. Jede okuläre Struktur kann betroffen sein: v. a. das neuroophthalmologische System, die Weichteile und das vordere Augensegment, aber auch retinale Veränderungen wurden beschrieben. Zum einen wurden vaskuläre Prozesse beobachtet: Man vermutet, dass hämorheologische Veränderungen und vaskuläre Turbulenzen zu Gefäßtortuositas, Exsudaten, Mikroaneurysmen, Cotten-Wool-Spots, Blutungen und Sehnervenschwellungen führen [1]. Außerdem kann es, wie hier beschrieben, zu einer Gammopathie-Makulopathie oder Paraproteinämie-Makulopathie mit seröser Makulaabhebung, intraretinalen Zysten und Exsudaten kommen [5]. In Fallserien wurde gezeigt, dass die Blut-Nerven-Schranke bei Patienten mit Gammopathien reduziert ist. Es wird angenommen, dass die Infiltration der neurosensorischen Netzhaut und des subretinalen Raums durch einen Exzess an Immunglobulinen einen osmotischen Druck in Richtung des Extrazellularraums auslöst, der zu einer erhöhten sub- und intraretinalen Transsudation führt. Wenn die Pumpfunktion des retinalen Pigmentepithels überstiegen wird, resultieren ein Makulaödem und eine seröse Makulaabhebung [1, 2]. In einer Studie konnte elektronenmikroskopisch und immunhistochemisch gezeigt werden, dass Leichtketten sich in der Bruch-Membran und in der inneren Chorioidea ablagern. Dies kann die Obstruktion der Choriokapillaris und eine Dysfunktion des retinalen Pigmentepithels zur Folge haben, wodurch wiederum die Phagozytose der Photorezeptoraußensegmente beeinträchtigt wird und hyperautofluoreszentes Material subretinal akkumuliert [3]. Auch das Auftreten des Pachychoroids wird der Ablagerung der Leichtketten zugeschrieben [3].

**Diagnose:** Das klinische Bild und die Fluoreszeinangiographie zeigten eine Makulopathie mit seröser Abhebung der neurosensorischen Netzhaut, intraretinaler Flüssigkeit, pigmentierte Ablagerungen und eine milde Angiopathie

Die Behandlung bei okulärer Beteiligung ist nicht standardisiert. Therapeutisch werden Chemotherapie, systemische oder intravitreale Kortisontherapie, Plasmapherese und intravitreale Anti-VEGF-Injektionen je nach klinischer Präsentation und auf Grundlage von Fallbeispielen durchgeführt. Die Ergebnisse variieren deutlich. In unserem Fall waren nach systemischer Steroidtherapie eine subjektive und objektive Besserung des Befundes zu verzeichnen.

## Fazit für die Praxis

- Manifestationen des multiplen Myeloms können in seltenen Fällen am Auge auftreten.
- Jede okuläre Struktur kann betroffen sein.
- Das Auftreten okulärer Veränderungen ist nicht zwangsläufig mit einer hohen Leichtketteninfiltration assoziiert.
- Die Therapie ist nicht standardisiert.
- Eine engmaschige Kontrolle der Patienten ist empfehlenswert.
- Mehr Studien sind wünschenswert.
